# Complete loss of the MHC II pathway in an anglerfish, *Lophius piscatorius*

**DOI:** 10.1098/rsbl.2019.0594

**Published:** 2019-10-09

**Authors:** Arseny Dubin, Tor Erik Jørgensen, Truls Moum, Steinar Daae Johansen, Lars Martin Jakt

**Affiliations:** Genomics group, Faculty of Biosciences and Aquaculture, Nord University, 8049 Bodø, Norway

**Keywords:** major compatibility complex, teleost, anglerfish, *Lophius*, gene loss, genome

## Abstract

Genome studies in fish provide evidence for the adaptability of the vertebrate immune system, revealing alternative immune strategies. The reported absence of the major compatibility complex (MHC) class II pathway components in certain species of pipefish (genus *Syngnathus*) and cod-like fishes (order Gadiformes) is of particular interest. The MHC II pathway is responsible for immunization and defence against extracellular threats through the presentation of exogenous peptides to T helper cells. Here, we demonstrate the absence of all genes encoding MHC II components (CD4, CD74 A/B, and both classical and non-classical MHC II *α*/*β*) in the genome of an anglerfish, *Lophius piscatorius*, indicating loss of the MHC II pathway. By contrast, it has previously been reported that another anglerfish, *Antennarius striatus*, retains all MHC II genes, placing the loss of MHC II in the *Lophius* clade to their most recent common ancestor*.* In the three taxa where MHC II loss has occurred, the gene loss has been restricted to four or five core MHC II components, suggesting that, in teleosts, only these genes have functions that are restricted to the MHC II pathway.

## 1. Introduction

The vertebrate adaptive immune system that generates diversity through genetic recombination appears to have evolved in the common ancestor of the jawed vertebrates (gnathostomes) [1]. Although this system increased in complexity with gnathostome evolution, it is thought that the acquisition of all required cellular processes, tissues and genes happened relatively quickly as most components are present across all jawed vertebrates [1]. T-cell receptors (TCR), B-cell receptors (BCR) and the major histocompatibility complex (MHC) classes I and II are all present throughout the gnathostome lineages, from the Chondrichthyes to terrestrial vertebrates [1]. Although the specific sites of haematopoiesis vary, homologous tissues and organs including the thymus and spleen are also present across the gnathostomes [1–4].

An intact adaptive immune system has been found in almost all vertebrate species that have had their genomes sequenced, but recent work has demonstrated the loss of components of the adaptive immune system in the elephant shark, pipefish, coelacanth and the entire Gadiformes order [5–10]. These observations demonstrate an unexpected plasticity of adaptive immunity.

The teleost order Lophiiformes (Anglerfishes) harbours at least 321 living species, approximately half of which express some degree of sexual parasitism [11]. In these species, males attach to the females either temporarily or permanently. In extreme cases, this leads to fusion of male and female circulatory systems [12]. Why this fusion does not result in tissue rejection is unknown, but suggests a specialized adaptive immune system. Phylogenetic inference based on sequencing data and morphology has concluded that male sexual parasitism within Lophiiformes must have multiple origins [11,13,14], suggesting a common selective pressure or a shared genetic predisposition.

Here, we present two independently obtained draft genomes of an anglerfish, *Lophius piscatorius*, and show that it has lost all components of the MHC II arm of the adaptive immune system. The MHC II pathway is known to be involved in allogenic rejection [15] and our observations suggest that loss of MHC II may have contributed to the immune tolerance observed in sexually parasitic anglerfish species.

## 2. Material and methods

### (a) Sample collection, DNA isolation and sequencing

Samples from two *L. piscatorius* individuals (referred to as BF1 and BF2) were collected in the Bodø coastal waters, Nordland County in collaboration with local fishermen. BF1 skeletal muscle and BF2 kidney were used for subsequent total DNA isolation, library preparation and sequencing. BF1 total DNA sequencing was performed using Illumina MiSeq and SOLiD 5500 technologies (sequence depth: 24×). BF2 total DNA was sequenced by Dovetail Genomics, USA on an Illumina HiSeq X instrument (sequence depth: 150×) as a service. The Illumina libraries were 300 bp paired-end reads with 600 bp insert size for the MiSeq, and 150 bp paired-end reads with 350 bp insert size for the HiSeq.

### (b) Bioinformatic analysis

The raw reads were trimmed from adapters and low-quality bases using Cutadapt [16]. Only Illumina data were used for the assemblies. Prior to assembly, overlapping read pairs were merged using FLASH (v. 1.2.11) [17]. Final assemblies were constructed with SPAdes (v. 3.10.0) [18]. Basic assembly statistics were calculated with QUAST (v. 4.4.1) [19] and gene-space completeness assessed using BUSCO (v. 2.0) [20] with the actinopterygii dataset (odb9).

**Figure 1.**
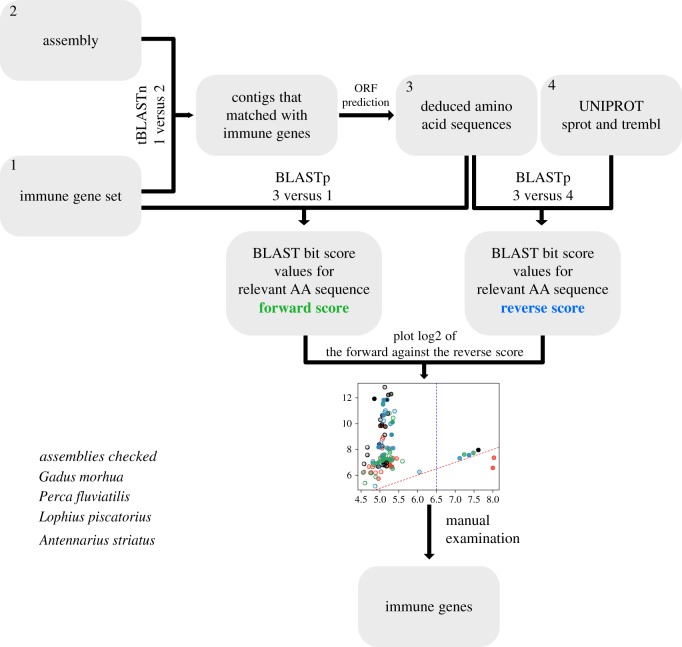
Outline of the gene mining process. Sequence inputs and outputs are shown as boxes with processes indicated by connecting arrows. BLAST inputs are numbered to indicate what was used as a query and subject (denoted as *query(number)* versus *subject(number)*). (Online version in colour.)

MHC genes were identified using methods similar to those used in [10] (figure 1). Briefly, a set of adaptive immune system-related protein sequences (bait-sequences) were used to identify contigs containing potential orthologues. Genes and open reading frames (ORFs) were predicted from these contigs and aligned both to the bait-set and sequences within the UniProt database to separate orthologues from non-orthologous genes containing homologous sequences. The resulting alignment scores were visualized and identities of candidate orthologues manually confirmed by inspection of alignments and annotations.

We performed this analysis on our *L. piscatorius* assemblies as well as on assemblies of *Antennarius striatus*, *Gadus morhua* and *Perca fluviatilis* [10]. If a gene was not identified in *L. piscatorius*, the unassembled reads (SOLiD and Illumina) were searched using tBLASTn. Matching reads were reassembled (CLC GW v. 11, QIAGEN, Aarhus, Denmark) and verified by reciprocal BLASTn against NCBI nr.

More detailed descriptions are provided in the electronic supplementary material.

## 3. Results

### (a) Genome assembly

The resulting *L. piscatorius* assemblies contained 664 (BF1) and 724 (BF2) megabases with N50 values of 6.9 kb and 108 kb, respectively. We used the BUSCO [20] actinopterygii set of 4584 conserved genes to estimate the gene-space completeness of these assemblies. We could detect at least 75% of these genes in both our assemblies (complete and fragmented), with 91.5% of complete genes identified in the BF2 assembly (electronic supplementary material, figure S1).

The gene space completeness of our assemblies is thus similar to that obtained for the *A. striatus* assembly (66.5% complete and 15.8% fragmented, electronic supplementary material, figure S1). Hence, our assemblies are comparable to or better than assemblies in [10] in terms of continuity, coverage and gene-space completeness (electronic supplementary material, figure S1).

### (b) Adaptive immune system genes in *L. piscatorius*

**Figure 2.**
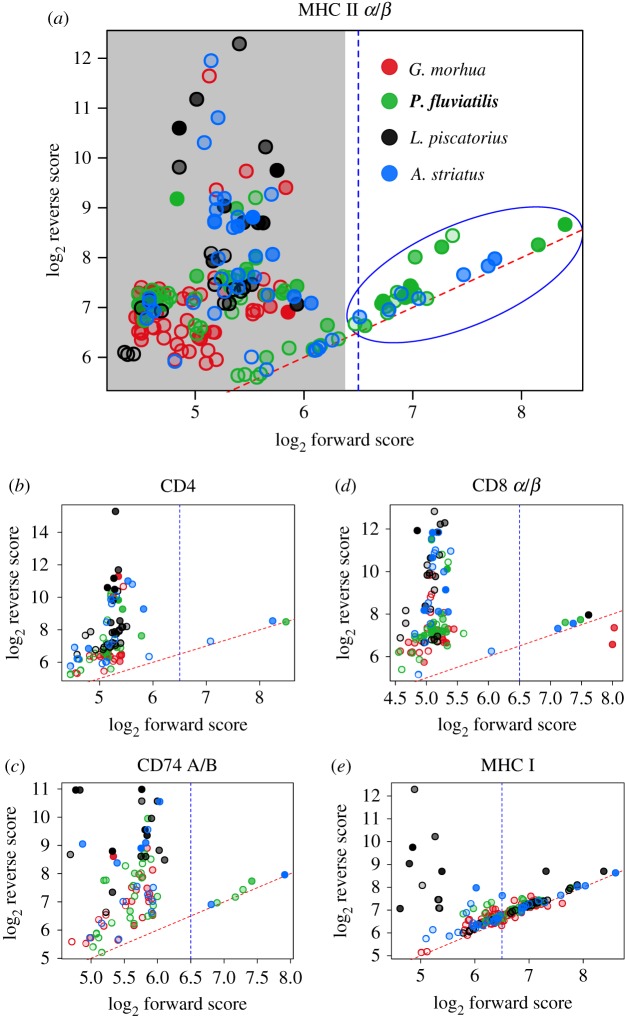
Identification of MHC I and MHC II pathway orthologues. Illustration of identification criteria (*a*). The alignment scores of putative orthologues against the initial bait set (forward score, *X*-axis) plotted versus scores against the UniProt database (reverse score, *Y*-axis). Grey shading indicates hits that were dismissed from further analysis, as they align better to genes not belonging to the MHC pathways. Alignments of short but highly conserved gene fragments can have high alignment scores without indicating functional orthologues; such points are shown with a high transparency by scaling the fill transparency by the ratio of alignment to subject (UniProt) length. Orthologues should have values closer to *Y* = *X* indicated by the dashed red line. Candidate orthologues shown within the blue ellipse appear as outliers, and can be identified by a forward score threshold indicated by the dashed blue line. Plots for the MHC II components (*a*–*c*) lack candidate orthologues for *Lophius piscatorius* (black) and *Gadus morhua* (red), whereas candidate orthologues are evident in all species for the MHC I genes (*d*,*e*). Results for the α/*β* chains of CD8 and MHC II, and the CD74 A/B genes are shown combined.

We used tBLASTn with a set of adaptive immune system genes to identify orthologous genes in *L. piscatorius* as well as in species previously reported to either have (*A. striatus*, *P. fluviatilis*) or lack (*G. morhua*) genes coding for MHC II components. Candidate orthologues were readily observed for all MHC I genes in all species. By contrast, we were unable to identify genes coding for CD4, CD74 A/B, MHC II *α*/*β* in either *L. piscatorius* or *G. morhua* assemblies (figure 2 and table 1). We repeated this analysis using an extended bait set including the non-classical MHC II *α*/*β* lineages [21]; this too failed to find any candidate orthologues in *L. piscatorius*. Similarly to the MHC I components we were also able to clearly identify orthologues of 22 out of 23 additional genes that have functions in the adaptive immune system (electronic supplementary material, figure S6, ST2).

**Table 1.** Number of candidate orthologues identified after forward/reverse screening (see §2) and manual inspection of the plots (figure 2). Numbers in brackets indicate individual hits after the forward score threshold was applied, but before manual examination of UniProt IDs identified unrelated genes.

gene	*Gadus morhua*	*Perca fluviatilis*	*Lophius piscatorius*	*Antennarius striatus*
CD4	**0**	1	**0**	2
CD74 A/B	**0**	4	**0**	2
MHC II *α*/*β*	**0**	21 (22)	**0**	6 (7)
CD8 *α*/*β*	2	2	1*	2
MHC I	49	34 (35)	18 (19)	12 (13)

*Predicted sequence appears as a fusion protein of *α* and *β* chains.

To confirm the absence of MHC II orthologues in *L. piscatorius* we also searched for short sequences in the unassembled reads that could be aligned with the missing genes. Using tBLASTn, we identified 18 and 62 reads from BF1 and BF2, respectively, which aligned with an MHC II *β* subunit. To locate the position of these potential MHC II sequences, the matching reads were assembled into contigs and mapped back to the original assemblies. This identified a region of approximately 300–480 bp in length present in both assemblies. When translated, the predicted reading frame was interrupted by multiple stop codons (electronic supplementary material, figure S2), indicating that the fragment represents a remnant of an MHC II *β* chain gene. Hence, we conclude that the MHC II pathway is absent in *L. piscatorius*.

To confirm the presence of genes syntenic to CD4 and CD74 in *L. piscatorius*, we identified contigs containing these genes [5] and aligned them to the stickleback (*Gasterosteus aculeatus*) loci (electronic supplementary material, figure S7). All highly conserved genes were identified in either a single (CD74) or three (CD4) contigs and the gene predictions lying within these contigs aligned both in terms of direction and order. CD74 in *L. piscatorius* seems to have been lost through a deletion of a region lying between ndst1a and SCL35A4 that has removed both CD74 and almost all intergenic space. For CD4, we were unable to identify a contig spanning the expected CD4 position; nevertheless, our analysis confirms the presence of the expected syntenic genes within our assembly.

### (c) MHC II in *A. striatus*

*Antennarius striatus* has been reported to contain both MHC I and MHC II pathway genes [10]. Since both *A. striatus* and *L. piscatorius* are members of the Lophiiformes order, we considered the possibility that the identification of *A. striatus* MHC II orthologues could have resulted from cross-contamination of the sample. Although we did observe the presence of cross-contaminating mitochondrial sequences from distantly related teleost taxa, the relative sequencing depth of contaminant and *A. striatus* sequences, combined with the unimodal depth distribution preclude the possibility that the MHC II sequences were derived from contaminant DNA fragments (electronic supplementary material, figure S4). Our observations thus confirm the presence of MHC II, while at the same time highlighting the potential of cross-contamination leading to confounding results.

## 4. Evolutionary considerations

Of the 81 teleosts examined so far, only members of the Gadiformes order (27 species sequenced), pipefishes (*Syngnathus typhle and S. scovelli*) and now *L. piscatorius* lack a functional MHC II pathway [5,6,9,10]. Notably, all these species lack CD4 and both MHC II *α* and *β* chains (classical and non-classical molecules). In addition, CD74 A/B has been completely lost in *L. piscatorius* and the Gadiformes order. Nevertheless, all contain a complete set of MHC I pathway components [6,9,10] (figure 2; electronic supplementary material, figure S6). In contrast to terrestrial vertebrates, teleost MHC II *α* and *β* often occupy multiple loci on different chromosomes [22]. This means that loss of the complete MHC II pathway requires multiple independent gene deletions; e.g. the loss of MHC II in *A. striatus* would require around 10 deletions (table 1). This suggests that loss of any critical MHC II pathway component leads to the loss of remaining core parts and argues that genes lost in these species are unlikely to have functions outside of MHC II in teleosts.

By contrast, genes such as CIITA, which is conventionally thought to be a specific activator of MHC II gene expression [23], is likely to have roles outside the MHC II system because in the absence of neo-functionalization, it would have no function after MHC II loss. Hence, our results are consistent with reports indicating an additional role of CIITA in the regulation of MHC I expression [24–26].

Sexual parasitism in anglerfish suggests some form of specialized immune system allowing for allogenic tolerance between fused individuals. Although CD8+ cytotoxic lymphocytes are thought to be the primary effectors of allogenic rejection, it is clear that MHC II components have both enabling and effector functions [15], and a long line of publications show that repression of MHC II components can contribute to immune tolerance or alleviate immune rejection [27–30]. Hence, it is tempting to speculate that the loss of MHC II in the Lophiodei suborder is not restricted to *Lophius* species and has played an enabling role in the development of sexual parasitism.

Most Lophiiformes phylogenies place the Lophioidei suborder at the most basal position in the anglerfish taxonomy, followed by Antennarioidei [11,14,31,32]. If Lophioidei is basal, then MHC II loss is likely to be specific to the Lophioidei suborder since *A. striatus* clearly possesses all MHC II components. That would also mean that our observations are unlikely to be relevant to sexual parasitism in other anglerfish clades. However, inferences of higher-order taxonomies are still fraught with difficulty, exemplified by the fact that the phylogenetic topology of the taxa involved based on mitochondrial DNA is subject to change depending on the choice of outgroup [11,13] (electronic supplementary material, figure S5). Exploring the presence and absence of MHC II genes in other anglerfish species can thus provide a test of the conventional phylogeny, as well as the likelihood of MHC II loss being one of the enabling adaptations preventing intra-species tissue rejection.

## 5. Conclusion

The classical MHC II components are responsible for the presentation of exogenous peptides to T helper cells and constitute an important part of the gnathostome adaptive immune system. Here, we report two draft genome assemblies of *L. piscatorius* and demonstrate a complete loss of the classical MHC II pathway in this species. The finding of a third taxon that lacks MHC II function corroborates the dispensability of MHC II in teleosts, and suggests that the genes lost in all three clades have no function outside of the MHC II.

## Supplementary Material

Supplementary material

## Supplementary Material

Predicted peptide sequences

## Supplementary Material

Scripts

## Supplementary Material

R scripts and functions.

## Supplementary Material

Top UniProt matches for hypothetical orthologues.

## Data accessibility

Sequences have been deposited to public database Dryad Digital Repository: https://doi.org/10.5061/dryad.4dc007q [33] and GenBank (SRR8809741 and SRR8801493).
