# Supplementary material for: Complete loss of the MHC II pathway in an anglerfish, Lophius piscatorius
Source: Biol Lett. 2019 Oct 9;15(10):20190594. doi: 10.1098/rsbl.2019.0594 (PMC6832177; doi:10.1098/rsbl.2019.0594)
Supplement: Supplementary material [file rsbl20190594supp1.pdf]

Electronic supplementary material from:

Complete loss of the MHC II pathway in an anglerfish, Lophius piscatorius

Arseny Dubin, Tor Erik Jørgensen, Truls Moum, Steinar Daae Johansen and Lars Martin Jakt

Genomics group, Faculty of Biosciences and Aquaculture, Nord University, 8049 Bodø, Norway

Author for correspondence:
Lars Martin Jakt
lars.m.jakt@nord.no

S1. Assembly statistics

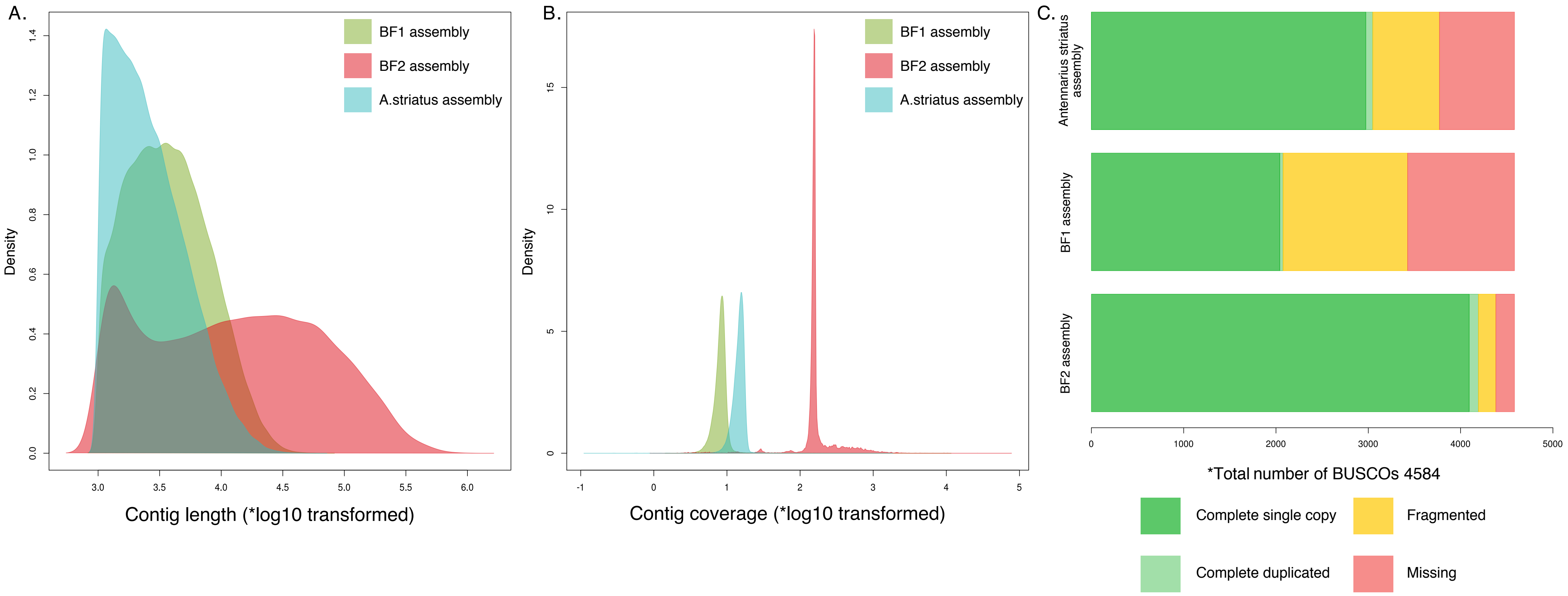

S2. Sequences from the BF1 and BF2 assemblies that could be aligned to MHC II

A.

BF1 MHC class II fragment. Frame -3.

```
ctatcctaaacaaatcaaatgggcaggatgtgacctcagctgtcatttttttctgaagtgttg
  I  L  N  K  S  N  G  Q  D  V  T  S  A  V  I  F  S  E  V  L
cctgatgtggaatagtactgccagattgagtcttacctggagtacatgccaacccctgga
 P  D  V  E  *  Y  C  Q  I  E  S  Y  L  E  Y  M  P  T  P  G
gcataaattacatgcatggataaacacctcagcctaccaaaacaagtgctttgagtctgg
 A  *  I  T  C  M  D  K  H  L  S  L  P  K  Q  V  L  *  V  W
ggcaaatttgcaattgtaatacaggatgagactatggacgatataaaaggagattaattt
 G  K  F  A  I  V  I  Q  D  E  T  M  D  D  I  K  G  D  *  F
ccctcctcatgtctgtagcaacatctcttcctggatcagagaggattataattattggaa
 P  S  S  C  L  *  Q  H  L  F  L  D  Q  R  G  L  *  L  L  E
caatttaactagtatttattttttgtcttagttagcactggcttcatctaatacaagtaaa
 Q  F  N  *  Y  L  F  L  S  *  L  A  L  A  S  S  N  T  S  K
atgcctggcgcagtcatcatataactgcaacttaagaaaaatattgttcttgtgctcatt
 M  P  G  A  V  I  I  *  L  Q  L  K  K  N  I  V  L  V  L  I
tttaacagtataagttgtgattcctgtggaatatgaccttgcagctgatgacaaaattat
 F  N  S  I  S  C  D  S  C  G  I  *  P  C  S  *  *  Q  N  Y
tatca
 Y
```

BF2 MHC class II fragment. Frame +3.

```
atctctttgtgctcctgcttattttcttcatctctcttgctatcccaatacttatttgctaa
   L  F  V  L  L  L  I  F  F  I  S  L  A  I  P  I  L  I  C  *
cttaatccatgcttgtgtgcaaagcctataaatactatcctaaacaaatcaaatgggcag
 L  N  P  C  L  C  A  K  P  I  N  T  I  L  N  K  S  N  G  Q
gatgtgacctcagctgtcatttttttctgaagtgttgcctgatgtggaatagtactgccag
 D  V  T  S  A  V  I  F  S  E  V  L  P  D  V  E  *  Y  C  Q
attgagtcttacctggagtacatgccaacccctggagcataaattacatgcatggataaa
 I  E  S  Y  L  E  Y  M  P  T  P  G  A  *  I  T  C  M  D  K
cacctcagcctaccaaaacaagtgctttgagtctggggcaaatttgcaattgtaatacag
 H  L  S  L  P  K  Q  V  L  *  V  W  G  K  F  A  I  V  I  Q
ga
```

B.

CLUSTAL O(1.2.4) alignment (*stop codons removed)

```
bf1      -------------------------------ILNKSNGQDVTSAVIFSEVLPDVEYCQIE 29
bf2      LFVLLLIFFISLAIPILICLNPCLCAKPINTILNKSNGQDVTSAVIFSEVLPDVEYCQIE 60
                                        *****************************

bf1      SYLEYMPTPGAITCMDKHLSLPKQVLVWGKFAIVIQDETMDDIKGDFPSSCLQHLFLDQR 89
bf2      SYLEYMPTPGAITCMDKHLSLPKQVLVWGKFAIVIQ------------------------ 96
         ************************************

bf1      GLLLEQFNYLFLSLALASSNTSKMPGAVIILQLKKNIVLVLIFNSISCDSCGIPCSQNYY 149
bf2      ------------------------------------------------------------ 96
```

C.

BF1 MHC class II fragment. Top 2 BLASTx hits at NCBI Nr

PREDICTED: rano class II histocompatibility antigen, A beta chain-like isoform X2 [Lates calcarifer]
Sequence ID: XP_018527094.1 Length: 266 Number of Matches: 1

Range 1: 151 to 213 GenPept Graphics — Next Match — Previous Match

Score	Expect	Method	Identities	Positives	Gaps	Frame
68.6 bits(166)	1e-10	Compositional matrix adjust.	34/63(54%)	44/63(69%)	0/63(0%)	-3

```
Query  470  NGQDVTSAVIFSEVLPDVE*YCQIESYLEYMPTPGA*ITCMDKHLSLPKQVL*VWGKFAI  291
            NGQ+VTSAV  S+ +PD + Y QI SYLEY PTPG  ITCM +HL+L + +L VW  F +
Sbjct  151  NGQEVTSAVSSSDAMPDGDWYYQIHSYLEYTPTPGETITCMVQHLTLSEPMLQVWDPFLL  210

Query  290  VIQ  282
              +
Sbjct  211  AAE  213
```

PREDICTED: rano class II histocompatibility antigen, A beta chain-like isoform X1 [Lates calcarifer]
Sequence ID: XP_018527088.1 Length: 299 Number of Matches: 1

Range 1: 184 to 246 GenPept Graphics — Next Match — Previous Match

Score	Expect	Method	Identities	Positives	Gaps	Frame
68.6 bits(166)	1e-10	Compositional matrix adjust.	34/63(54%)	44/63(69%)	0/63(0%)	-3

```
Query  470  NGQDVTSAVIFSEVLPDVE*YCQIESYLEYMPTPGA*ITCMDKHLSLPKQVL*VWGKFAI  291
            NGQ+VTSAV  S+ +PD + Y QI SYLEY PTPG  ITCM +HL+L + +L VW  F +
Sbjct  184  NGQEVTSAVSSSDAMPDGDWYYQIHSYLEYTPTPGETITCMVQHLTLSEPMLQVWDPFLL  243

Query  290  VIQ  282
              +
Sbjct  244  AAE  246
```

BF2 MHC class II fragment. Top 2 BLASTx hits at NCBI Nr

PREDICTED: rano class II histocompatibility antigen, A beta chain-like isoform X2 [Lates calcarifer]
Sequence ID: XP_018527094.1 Length: 266 Number of Matches: 1

Range 1: 151 to 213 GenPept Graphics — Next Match — Previous Match

Score	Expect	Method	Identities	Positives	Gaps	Frame
68.2 bits(165)	2e-11	Compositional matrix adjust.	34/63(54%)	44/63(69%)	0/63(0%)	+3

```
Query  114  NGQDVTSAVIFSEVLPDVE*YCQIESYLEYMPTPGA*ITCMDKHLSLPKQVL*VWGKFAI  293
            NGQ+VTSAV  S+ +PD + Y QI SYLEY PTPG  ITCM +HL+L + +L VW  F +
Sbjct  151  NGQEVTSAVSSSDAMPDGDWYYQIHSYLEYTPTPGETITCMVQHLTLSEPMLQVWDPFLL  210

Query  294  VIQ  302
              +
Sbjct  211  AAE  213
```

PREDICTED: rano class II histocompatibility antigen, A beta chain-like isoform X1 [Lates calcarifer]
Sequence ID: XP_018527088.1 Length: 299 Number of Matches: 2

Range 1: 184 to 246 GenPept Graphics — Next Match — Previous Match

Score	Expect	Method	Identities	Positives	Gaps	Frame
68.2 bits(165)	2e-11	Compositional matrix adjust.	34/63(54%)	44/63(69%)	0/63(0%)	+3

```
Query  114  NGQDVTSAVIFSEVLPDVE*YCQIESYLEYMPTPGA*ITCMDKHLSLPKQVL*VWGKFAI  293
            NGQ+VTSAV  S+ +PD + Y QI SYLEY PTPG  ITCM +HL+L + +L VW  F +
Sbjct  184  NGQEVTSAVSSSDAMPDGDWYYQIHSYLEYTPTPGETITCMVQHLTLSEPMLQVWDPFLL  243

Query  294  VIQ  302
              +
Sbjct  244  AAE  246
```

Range 2: 163 to 178 GenPept Graphics — Next Match — Previous Match — First Match

Score	Expect	Method	Identities	Positives	Gaps	Frame
28.1 bits(61)	2e-11	Compositional matrix adjust.	10/16(63%)	14/16(87%)	0/16(0%)	+2

```
Query  68   SMLVCKAYKYYPKQIK  115
            ++LVC AY +YPKQI+
Sbjct  163  AILVCSAYNFYPKQIQ  178
```

S3. Contaminant mitochondrial sequences in *A. striatus*

Contig identifier	Length (bp)	Match (identity)	Subject/query alignment coordinates
**utg7180000251100**	1749	Trachurus japonicus (AP003091.1) and Trachurus trachurus (AB108498.1) 98%	7223-5475/1-1749
**utg7180000189499**	3298	Trachurus japonicus (AP003091.1) 98%	10559-7263/1-3297
**utg7180002535071**	8275	Trachurus japonicus (AP003091.1 and AP003092.1) 97%	10559-16559/1-6000<br>1-2276/6001-8275
**utg7180002551981**	3238	Trachurus japonicus (AP003091.1 and AP003092.1) 94%	5430-2193/1-3238
**utg7180000416975**	1218	Decapterus maruadsi (KJ004518.1) 87.44%<br>Decapterus macarellus (KM986880.1) 86.86%	9937-11146/9-1218<br>9938-11147/9-1218
**utg7180002761404**	2531	Decapterus maruadsi (KJ004518.1) 91%	7861-5334/4-2531
**utg7180000037847**	2676	Coreoperca loona (KJ644781.1) 86.59%<br>Siniperca scherzeri (AP014527.1) 86.57%	203-2786/1-2609<br>202-2784/1-2609
**utg7180002123755**	13110	Emmelichthys struhsakeri (AP004446.1) 79.72%<br>Monodactylus argenteus (AP009169.1) 79.63%	2787-15652/59-12949<br>2786-15672/59-12972

S4. Coverage of MHC II and mitochondrial sequences in *A. striatus*

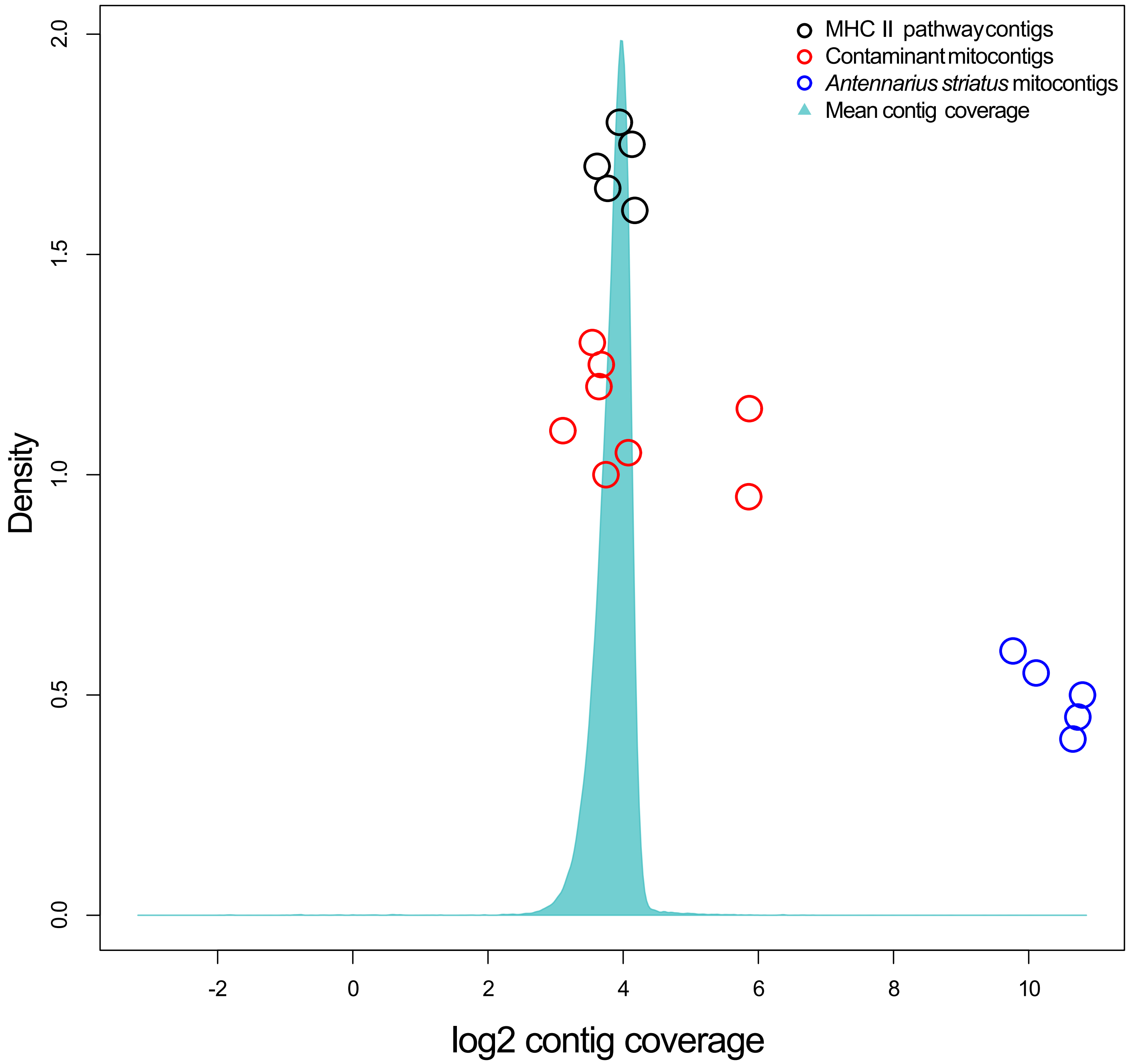

S5. Phylogenetic trees based on complete mitochondrial genome sequences of species in the Lophiiformes order

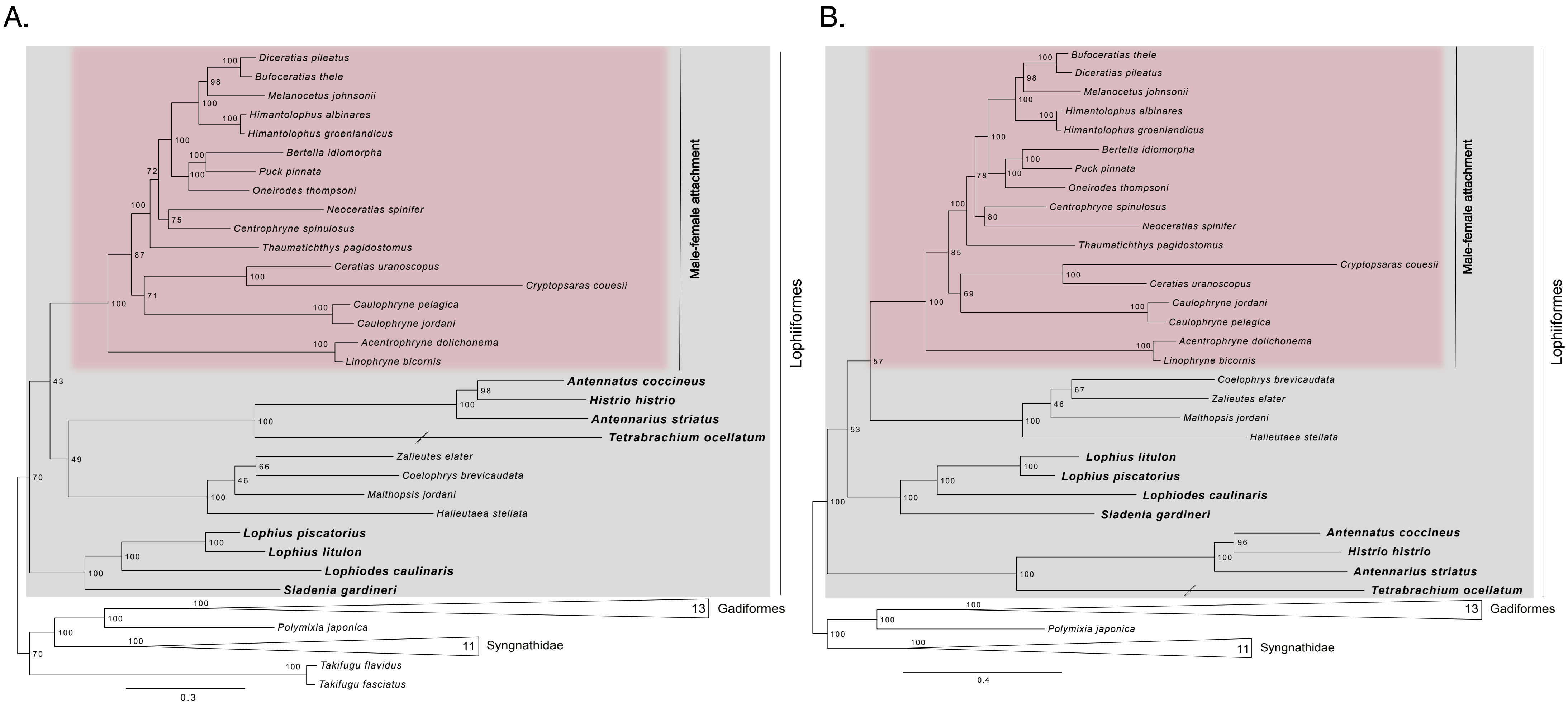

S6. Identification of immune gene orthologues, pages 6-11

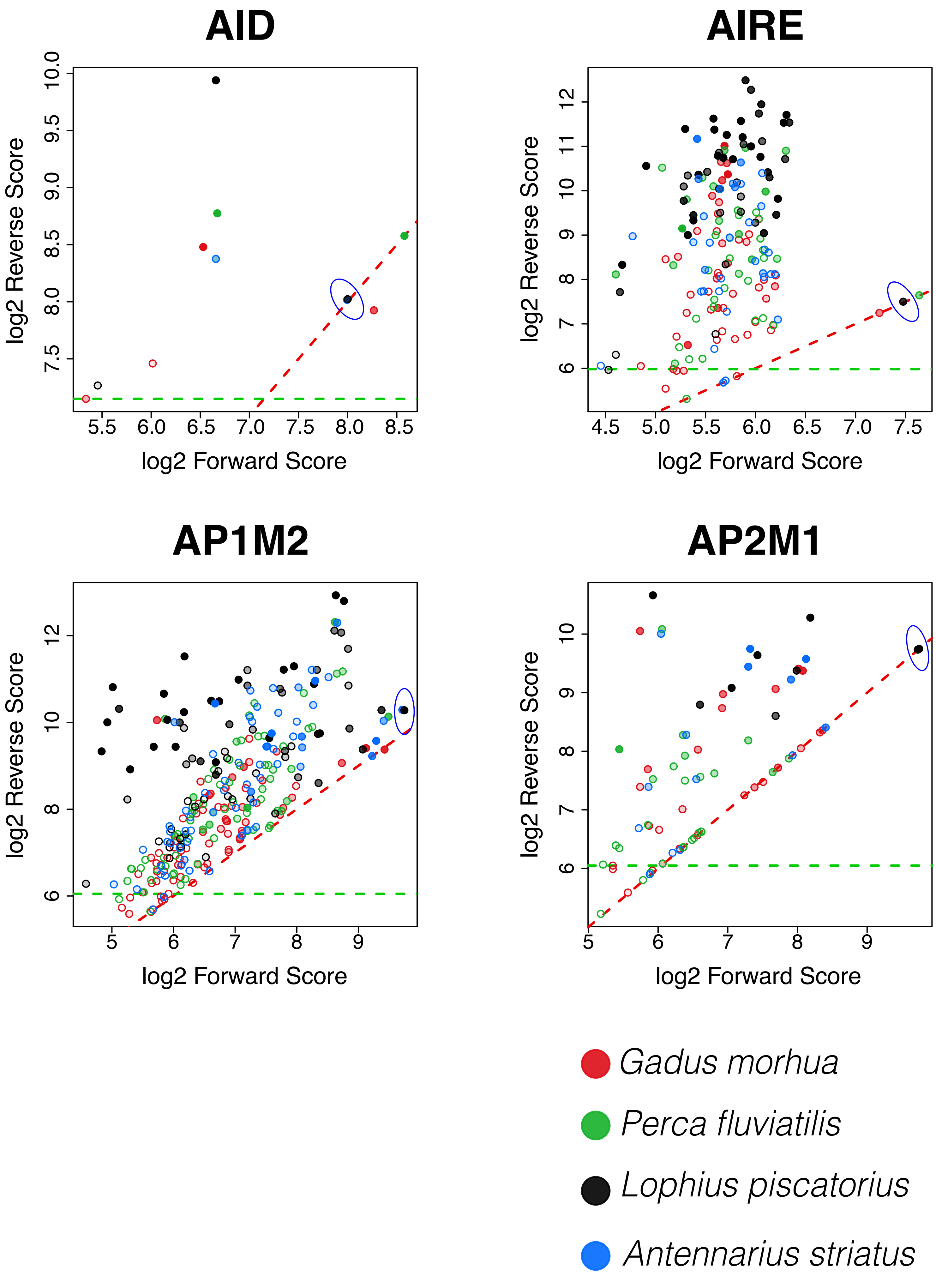

r. Identification of immune gene orthologues (Continued from p.6)

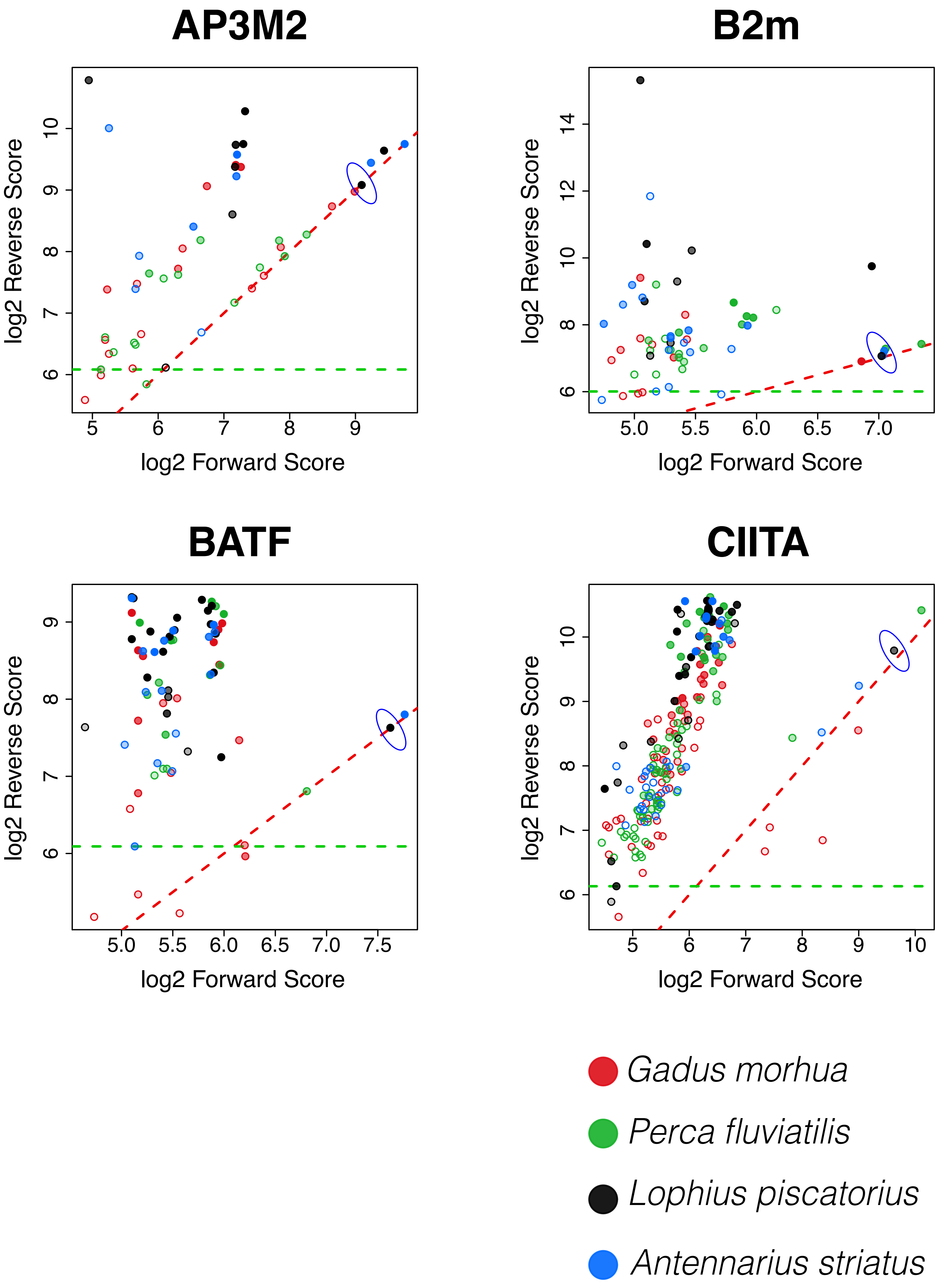

S6. Identification of immune gene orthologues (Continued from p.6)

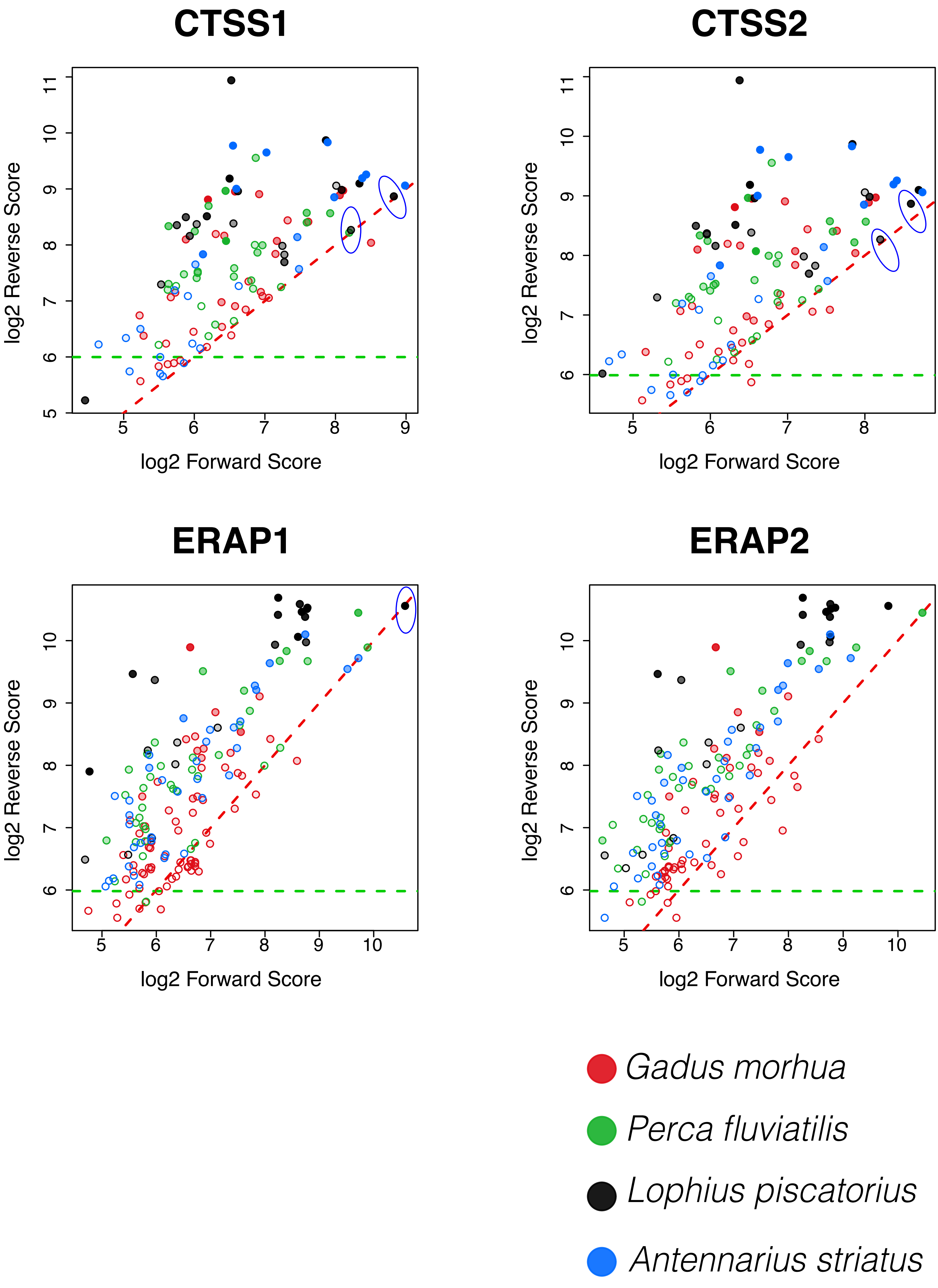

S6. Identification of immune gene orthologues (Continued p.6)

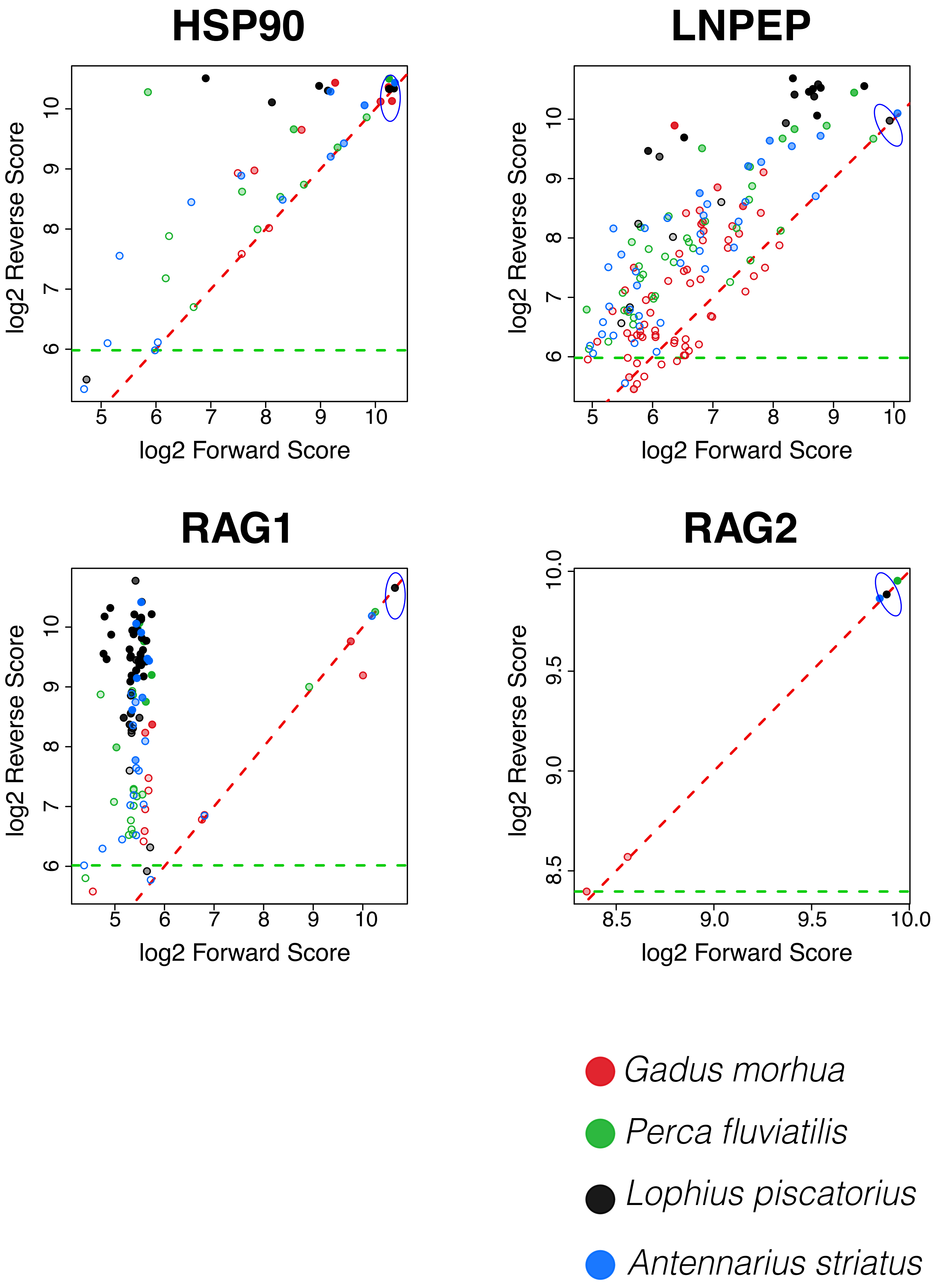

S6. Identification of immune gene orthologues (Continued p.6)

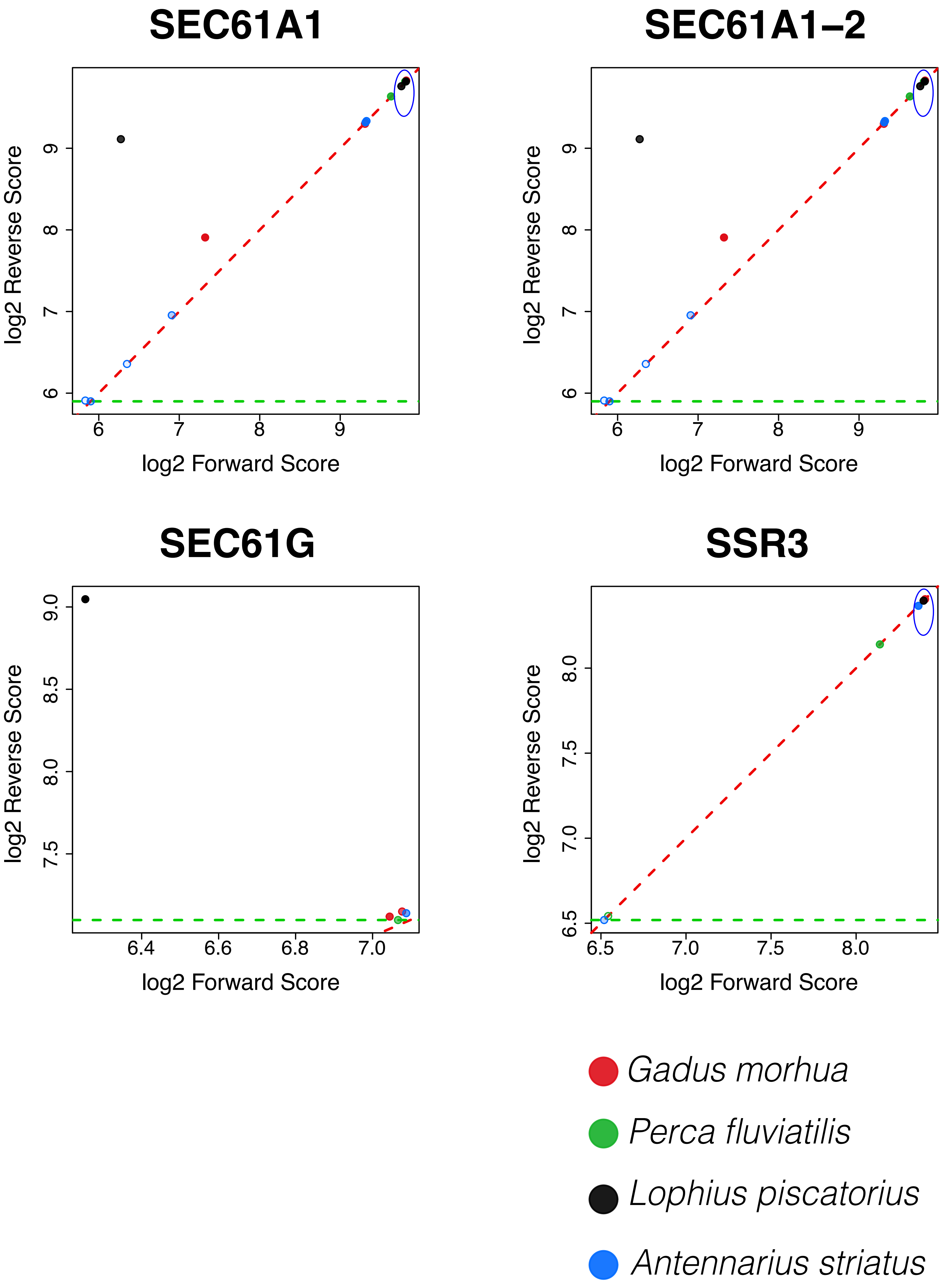

S6. Identification of immune gene orthologues (Continued p.6)

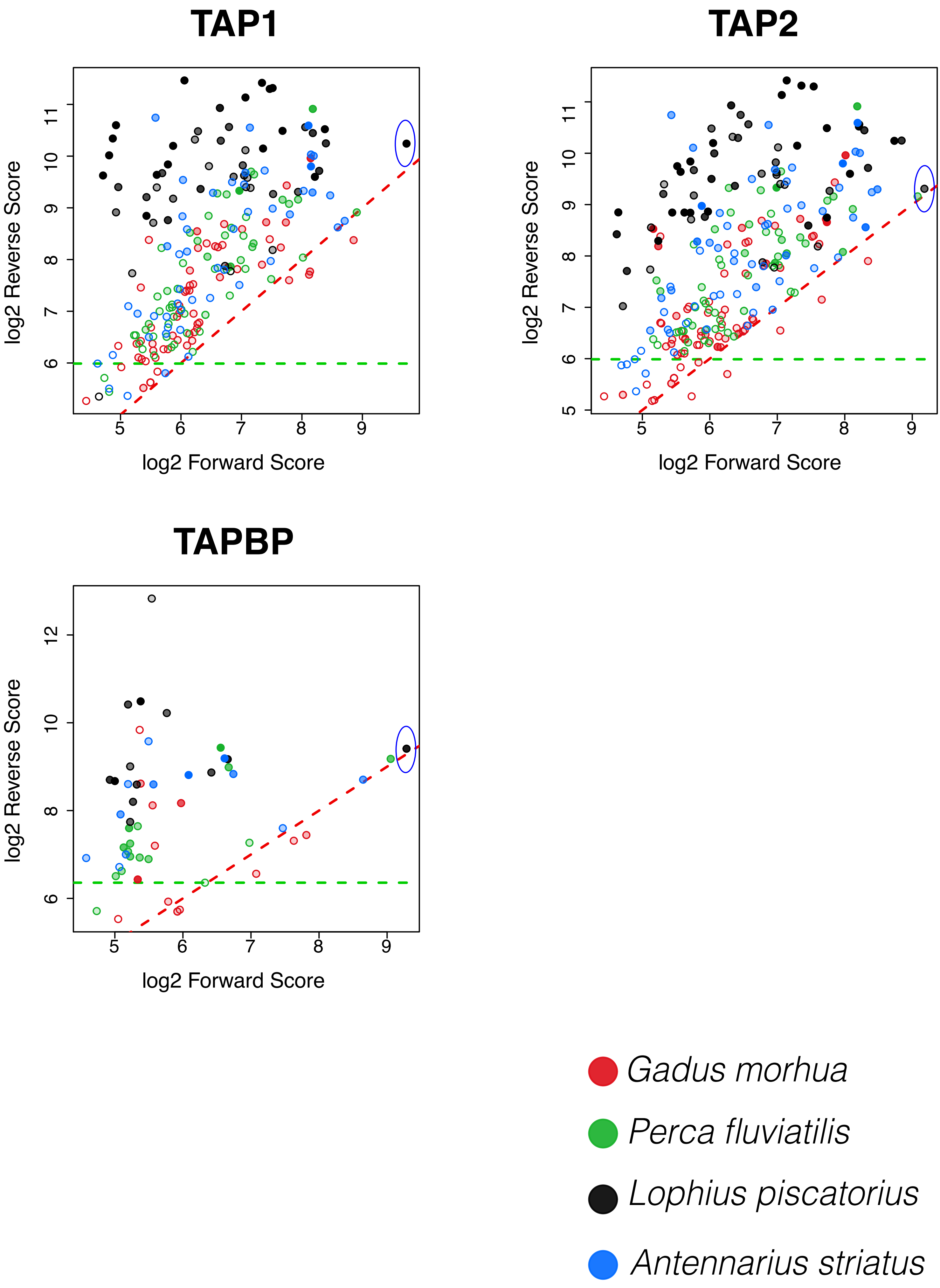

S7. Gene synteny for the CD74 and CD4 gene regions

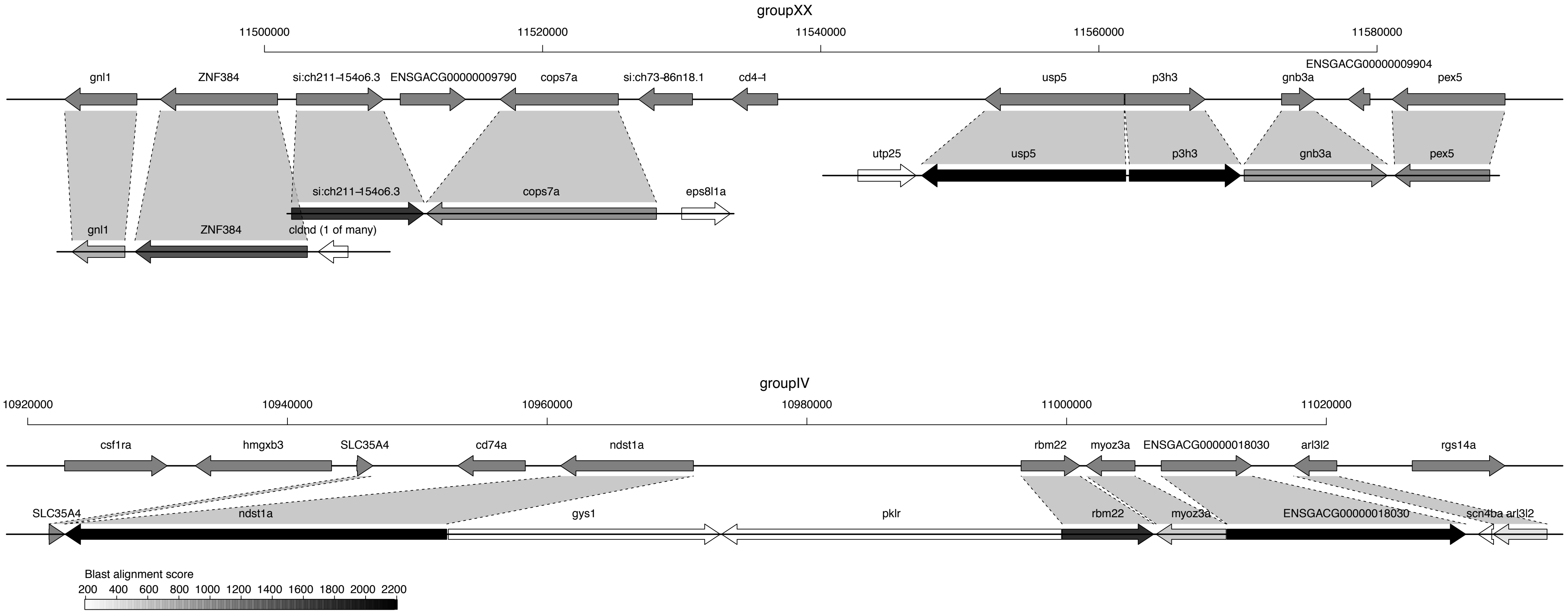

Supplementary figure legends

S1. Assembly statistics

Kernel density estimates of log (base 10) transformed contig length (A), coverage (B) and gene completeness (C) for the two *L. piscatorius* and the single *A. striatus* assembly.

S2. Sequences from the BF1 and BF2 assemblies that could be aligned to MHC II

A. Nucleotide and deduced amino acid sequences of the identified MHC II fragments. Residues shown in red mark the amino acids that were aligned by BLAST to MHC II β sequences.

B. Alignment of amino acid sequences from both assemblies (with stop codons removed) that aligned to MHC II β confirm that the same sequence was identified in both assemblies.

C. Top 2 BLASTx hits for the identified fragments. The figure shows direct screen-grabs from the NCBI BLAST web service.

S3. Contaminant mitochondrial sequences in *A. striatus*

Hits that we consider identifiable to a species level are marked with green. All sequences were identified with BLASTn and e-value threshold of 10. Hits shorter than a 1000 bp were discarded.

S4. Coverage of MHCII and mitochondrial sequences in *A. striatus*

Kernel density estimate of log2 transformed mean coverage for all contigs (cyan), mean coverage of MHC II containing contigs (black points) and mitochondrial contigs (red and blue points). Mitochondrial contigs identified as *A. striatus* sequences (blue points) were sequenced at 16 to 64 times the depth observed for contaminant mitochondrial sequences (red points). Each point represents one contig. Mean coverage for each contig was calculated by mapping quality trimmed reads to the assembly, converting bam to by-base coverage bed files and calculating the mean.

S5. Phylogenetic trees based on complete mitochondrial genome sequences of species in the Lophiiformes order

The internal branch ordering is dependent on the choice of outgroups, with the position of the Antennariodei and Lophiodei clades occupying the most basal position in A and B respectively.

The scale indicates the number of substitutions per site. The *Tetrabrachium ocellatum* branch length has been halved due to its extreme length. Node support values are bootstrap probabilities based on 500 iterations.

Phylogenetic relationships were inferred using a partitioned maximum likelihood analysis (with first, second and third codon positions, rRNA and tRNA as partitions) and a GTR GAMMA model as implemented in RaxML [1].

A. Tree created using *Polymixia japonica*, Gadiformes, Syngnathidae and Tetraodontiformes as the outgroups.

B. Tree created using *Polymixia japonica*, Gadiformes and Syngnathidae as the outgroups.

S6. Identification of immune gene orthologues. Pages 6-11

Illustration of identification criteria. Scores of alignments of putative orthologue sequences to the initial bait set (forward score, X-axis) plotted against scores obtained by alignment to sequences in the UniProt database (reverse score, Y-axis). The point fill transparency indicates the ratio of the alignment length to the length of the UniProt subject. Solid fills (alpha=1) correspond to full length alignments (i.e. the alignment covers the complete UniProt sequence). indicates relationship between the alignment length and length of the UniProt subject. Solid fill colour corresponds to 1/1 relationship. Orthologues should lie close to the Y=X line indicated by the dashed red line. The green dashed line shows the inferred e-10 e-value threshold. Points that we think represent orthologous sequences are marked with a blue ellipse. Peptide IDs corresponding to the selected points are collected in supplementary table 2, along with comments about the selection process., along with some comments about selection process. For additional information see *gene.hits.tsv* and *esm_pisc_pep.fasta*.

S7. Gene synteny for the CD74 and CD4 gene regions in *L.piscatorius* and *G. aculeatus*

The locations of orthologues to genes that are usually found in the CD4 and CD74 regions were identified in the BF2 assembly of *L. piscatorius* in the same manner as described above. To verify the identity of predicted genes we also aligned them to the NCBI nr database and manually inspected the resulting alignments. The synteny of the genes lying in the identified contigs was tested in *Gasterosteus aculeatus*. Genscan predicted peptides were blasted against *G. aculeatus* sequences (Ensembl *gasterosteus aculeatus* core 97.1). The top scoring alignment was taken as the gene identity for the genscan predictions and the matching contigs were aligned to their respective *G. aculeatus* loci using coordinates provided by Ensembl and the genscan predictions using a custom R-script. Top and bottom panel: CD4 and CD74 loci respectively. Genome positions in *G. aculeatus* are indicated by the scale bar; groupXX and groupIV are linkage group identifiers.

Shading of *L. piscatorius* gene predictions indicate the blast alignment score. Plots are to scale.

Supplementary Methods

****To find all scripts referred to in this ESM *see esm_scripts.txt***

Sequencing and genome assembly

The raw reads were trimmed from adapters and low quality bases using Cutadapt [2] with 25 as a quality threshold. Only Illumina data was used for the assemblies. Prior to assembly, overlapping read pairs were merged using FLASH (v1.2.11) [3]. Final assemblies were constructed with SPAdes (v3.10.0) [4] employing 6 kmer lengths (21, 33, 55, 77, 99, 127/103). Basic assembly statistics were calculated with QUAST (v4.4.1) [5] and gene-space completeness assessed using BUSCO (v2.0) [6] with the actinopterygii dataset (odb9). The trimmed reads were used to approximate the genome size with Jellyfish (v2.2.6) [7] and a suite of perl scripts (http://josephryan.github.com/estimate_genome_size.pl/).

Orthologue identification

In order to identify orthologues of adaptive immune system genes in *Lophius piscatorius* genome assemblies without the use of a predetermined e-value and bit score thresholds, we developed the strategy described below. Each step described was implemented in a short python (.py) or shell (.sh) script as indicated.

1. Identification of contigs that contain immune genes

We used a set of full-length amino acid sequences of 29 immune genes [8] and HSP90 from 10 species to search for orthologues in both our assemblies (BF1 and BF2). We also performed the same procedure for previously published draft genome assemblies of *Antennarius striatus*, *Gadus morhua and Perca fluviatilis* [8] as positive and negative controls to validate our strategy.

Sequences for the following species were obtained from Ensembl:

Danio rerio, Gadus morhua, Gasterosteus aculeatus, Tetraodon nigroviridis , Orytzias latipes, Oreochromis niloticus, Takifugu rubripes, Xiphophorus maculatus, Poecilia formosa, Astyanax mexicanus

Genes in the dataset:

1.AID	6.B2m	11.CIITA	16.HSP90	21.MHCIIa	26.SEC61A1-2	31.TAPBP
2.AIRE	7.BATF	12.CTSS1	17.CD74a	22.MHCIIb	27.SEC61G	
3.AP1M2	8.CD4	13.CTSS2	18.CD74b	23.RAG1	28.SSR3	
4.AP2M1	9.CD8a	14.ERAP1	19.LNPEP	24.RAG2	29.TAP1	
5.AP3M2	10.CD8b	15.ERAP2	20.MHCI	25.SEC61A1	30.TAP2	

To identify contigs containing candidate orthologues, we aligned the peptide sequences encoded by these genes to assemblies using tBLASTn (*manyfish_blast.py*). To reduce the false negative rate at this step we used a permissive e-value threshold of 1 (compared to the e-10 threshold usually used) but limited the number of target sequences to 50 and relied on post-filtering to remove incorrect matches.

Identifiers of contigs containing alignments to the seed genes were extracted from the BLAST output and split by assembly and gene into separate files (*process_blast.py*) which were used for downstream analyses.

2. Contig extraction and gene prediction

Selected contigs were subjected to gene prediction by Genscan [10], resulting in a set of amino acid sequences for each immune gene and matching contig. These sequence sets included both the amino acid sequences of orthologous immune genes and unrelated sequences located within the same contigs. To identify the orthologues, we used two further BLASTp screens which we refer to as Forward and the Reverse BLAST.

All predicted peptides from the BF2 *L. piscatorius* assembly (including non-immune peptides) sorted by gene can be found in the *esm_pisc_pep.fasta* file.

3. Forward BLAST

In order to provide alignment scores that could be compared to those in an extended blast against UniProt (step 4), we aligned amino acid sequence sets identified by Genscan in step 2 to the initial seed set (*forward_blast.sh*) using BLASTp. Again we used an e-value threshold of 1 and limited the number of target sequences to 50.

Peptide sequences aligning to their respective seed genes from step 1 were selected for further analyses (*filter_forward_blast.sh*). For example, peptides derived from contigs identified by tBLASTn with AIRE as a query were filtered to remove all peptides not aligned to AIRE.

We refer to the BLASTp bit score values obtained in this search as the Forward BLAST score.

4. Reverse BLAST

The majority of alignments obtained in the first rounds of blast with the seed set of immune genes are likely to involve proteins that are not orthologous, but which contain domains with some homology with seed set domains. Such sequences should align with better scores to their true orthologues, at least some of which we would expect to find within the UniProt database.

Hence, we aligned the candidate immune peptide sequences from step 2 to the UniProt KB database (*reverse_blast.sh*). Again, the e-value threshold was 1 and the number of target sequences in the output was limited to 50. This is similar to the rationale for reciprocal blast, and for this reason we refer to this step as reverse blast even though technically both step 3 and 4 are done in the same direction.

We refer to the BLASTp bit score values obtained in this search as the Reverse BLAST score.

5. Comparison of the Forward and Reverse BLAST scores

In theory, immune gene orthologues should align to the initial immune set (from step 1) and to the UniProt with similar bit scores, i.e. have similar Forward and Reverse scores, whereas non-orthologous sequences should be aligned with a higher score to their true orthologues present in Uniprot and hence have higher Reverse scores.

To determine whether it was possible to separate true orthologues by comparing forward and reverse scores we plotted forward against reverse (log)scores (*R script bl_revision.R, functions.R see functions_and_R_scripts.txt*). Since truncated orthologue sequences would still have similar forward and reverse scores (reflecting their identity), we also visualised the ratio of the alignment length to the Uniprot sequence length using alpha transparency values for points such that points reflecting alignments to non-truncated sequences (i.e. similar sequence length) appear as solid points.

6. Visual/manual examination of plots/hits

To verify the identity of candidate immune gene orthologues we used the *identify* function in R to examine the UniProt annotation of selected alignments. For most immune genes, orthologues were easily identifiable as they lied on/or very close to the forward = reverse score line and were aligned to a UniProt protein annotated as the desired immune gene orthologue. However, for some genes we observed multiple points on or close to this line (AP1M2, AP3M2, CTSS1/2), the UniProt annotation did not match with the selected gene (ERAP1, TAP1), or none of the points on the plot fitted our criteria (SEC61G, ERAP2). In this case, we chose several points that might represent an orthologue and examined their top 5 UniProt hits (*gene.hits.tsv*)

Supplementary Table 2

Gene name	Orthologous predicted protein	Comments
AID	NODE_3337_length_66067_cov_44.9838|GENSCAN_predicted_peptide_3|233_aa	Top scoring UniProt hit belongs to correct gene
AP2M1	NODE_326_length_249814_cov_47.6866|GENSCAN_predicted_peptide_14|1074_aa<br>NODE_6641_length_27025_cov_42.6987|GENSCAN_predicted_peptide_1|1026_aa	Top scoring UniProt hit of the first peptide and third of the second peptide belongs to correct gene
AP3M2	NODE_11858_length_6461_cov_171.208|GENSCAN_predicted_peptide_1|405_aa	Top scoring UniProt hit belongs to correct gene
B2m	NODE_26321_length_534_cov_183.369|GENSCAN_predicted_peptide_1|92_aa	Top scoring UniProt hit belongs to correct gene
BATF	NODE_6109_length_31083_cov_52.3689|GENSCAN_predicted_peptide_2|251_aa	Top scoring UniProt hit belongs to correct gene
CIITA	NODE_2303_length_93200_cov_43.342|GENSCAN_predicted_peptide_4|1556_aa	Top scoring UniProt hit belongs to correct gene
CTSS1	NODE_2178_length_97347_cov_44.3687|GENSCAN_predicted_peptide_7|470_aa	Top scoring UniProt hit belongs to correct gene
CTSS2	NODE_969_length_161682_cov_49.7592|GENSCAN_predicted_peptide_5|404_aa	Top scoring UniProt hit belongs to correct gene
HSP90	NODE_170_length_315068_cov_45.2866|GENSCAN_predicted_peptide_20|709_aa	Top scoring UniProt hit belongs to correct gene
LNPEP	NODE_1284_length_138026_cov_45.1581|GENSCAN_predicted_peptide_8|971_aa	Top scoring UniProt hit belongs to correct gene
RAG 1	NODE_1604_length_120776_cov_52.7006|GENSCAN_predicted_peptide_2|1015_aa	Top scoring UniProt hit belongs to correct gene
RAG 2	NODE_1604_length_120776_cov_52.7006|GENSCAN_predicted_peptide_3|533_aa	Top scoring UniProt hit belongs to correct gene
SEC61A1	NODE_148_length_331228_cov_45.7995|GENSCAN_predicted_peptide_18|494_aa	Top scoring UniProt hit belongs to correct gene
SEC61A1-2	NODE_4460_length_48594_cov_76.0295|GENSCAN_predicted_peptide_1|454_aa	Top scoring UniProt hit belongs to correct gene
SSR3	NODE_3618_length_61353_cov_45.4674|GENSCAN_predicted_peptide_2|296_aa	Top scoring UniProt hit belongs to correct gene
TAP2	NODE_4645_length_46251_cov_64.2593|GENSCAN_predicted_peptide_5|875_aa	Top scoring UniProt hit belongs to correct gene
TAPBP	NODE_11231_length_7753_cov_428.536|GENSCAN_predicted_peptide_1|456_aa	Top scoring UniProt hit belongs to correct gene
ERAP1	NODE_1839_length_110210_cov_47.4795|GENSCAN_predicted_peptide_7|886_aa	After examination, top UniProt hit belongs to correct gene
AIRE	NODE_2144_length_98088_cov_45.0458|GENSCAN_predicted_peptide_7|218_aa	Second UniProt hit and two others belong to correct gene
TAP1	NODE_39_length_479181_cov_44.6292|GENSCAN_predicted_peptide_18|1443_aa	Fusion prediction. Selected first part of the sequence
AP1M2	NODE_938_length_164357_cov_43.2764|GENSCAN_predicted_peptide_8|1716_aa	Fusion prediction. Selected last part of the sequence
ERAP2	Unclear orthology due to too many paralogous aminopeptidases.	?
SEC61G	See figure legend for detail.	Special case. See figure legend.

Supplementary Table 2

The table includes identifiers of the predicted peptides (column 2) that we consider to be orthologues to the target set of immune genes (column 1). Column 3 contains short comments on how this gene was identified. For most genes, the top blast hit lying on the X=Y line corresponded clearly to a UniProt protein annotated as the respective target gene. However, for some genes we had to examine the annotation of additional hits, due to non-informative description of the top hit, e.g. in the case of AIRE the top UniProt hit was described as Chromosome_15_SCAF14992. In addition, some predicted peptides combined products from two adjacent genes (Fusion prediction). For these genes the alignment coordinates had to be examined. The predicted protein sequences can be found in *esm_pisc_pep.fasta* and a summary of the blast output for selected points is provided in *gene.hits.tsv.*

SEC61G of *L. piscatorius* was a special case. Although SEC61G is a highly conserved gene, it is short and one exon primarily contains low-complexity sequence. This results in alignments to the second exon having low BLAST scores leading to its exclusion from the gene prediction and resulting in a truncated protein sequence. However, a manual examination of the BLAST output clearly demonstrated that complete sequence was aligned with a high sequence similarity (but low score). Similarly, running BLAST with '-dust no' provided the full alignment with a high alignment score. It is notable that SEC61G is one of the genes that Malmstrøm et al. failed to identify in a number of species.

7. Unassembled reads search

Protein sequences from genes for which we failed to identify *L. piscatorius* orthologues with the Forward/Reverse BLAST strategy were used in a tBLASTn search of the unassembled read pools. In this case, we included both Illumina and SOLiD reads. Reads that were aligned to the missing protein sequences were re-assembled with CLC Genomics Workbench. The resulting contigs were aligned to the NCBI nr database with BLASTn. If this approach failed to identify missing orthologues, we aligned selected unassembled reads to the NCBI nr database. After this, we reported orthologues that we failed to identify as actually missing.

Construction of phylogenetic trees

All sequences were obtained from genbank (see accession numbers in the section below). Then, mitogenomes were split by gene according to their annotations. First, each protein coding gene, each tRNA and rRNA were aligned separately with T-Coffee [9]. Then, alignments were trimmed from the ends, to remove end gaps and sequences were concatenated into new mitogenome sequences for all species. Datasets were partitioned by the first, second and third codon positions for protein coding genes, then rRNA and tRNA were put as separate partitions. To construct the trees we used RaxML [1] using the GTR GAMMA model with 500 rapid bootstrap (-f a option) iterations.

Sequences used to construct the trees:

Polymixiidae

NC_002648 *Polymixia japonica*

Tetraodontiformes

GQ409967 *Takifugu fasciatus*
KJ562276 *Takifugu flavidus*

Syngnathiformes

KJ184525 *Syngnathoides biaculeatus*
KU925872 *Syngnathus typhle*
KJ184524 *Solegnathus hardwickii*
AP012309 *Doryrhamphus japonicus*
AP013027 *Hippocampus histrix*
KJ184528 *Trachyrhamphus serratus*
KP861226 *Syngnathus schlegeli*
JX970973 *Hippocampus comes*
NC_010272 *Hippocampus kuda*
NC_022722 *Hippocampus erectus*
KJ139455 *Corythoichthys flavofasciatus*

Gadiformes

AP018148 *Gadiculus argenteus thori*
X99772 *Gadus morhua*
KC844053 *Lota lota*
NC_008225 *Ventrifossa garmani*
NC_015102 *Micromesistius poutassou*
NC_004377 *Physiculus japonicus*
NC_015094 *Pollachius virens*
NC_010122 *Arctogadus glacialis*
NC_015120 *Merluccius merluccius*
NC_010121 *Boreogadus saida*
NC_008224 *Trachyrincus murrayi*
NC_008222 *Bathygadus antrodes*
NC_008124 *Bregmaceros nectabanus*

Lophiiformes

AB282831	*Tetrabrachium ocellatum*
AB282828	*Antennarius striatus*
AP005977	*Halieutaea stellata*
AB282837	*Neoceratias spinifer*
AB282847	*Thaumatichthys pagidostomus*
AB282836	*Caulophryne pelagica*
AB282830	*Antennatus coccineus*
AB282841	*Bufoceratias thele*
AB282842	*Diceratias pileatus*
AB282827	*Sladenia gardineri*
AB282855	*Acentrophryne dolichonema*
AB282854	*Linophryne bicornis*
AB282840	*Himantolophus groenlandicus*
AB282829	*Histrio histrio*
AB282849	*Centrophryne spinulosa*
AB282839	*Himantolophus albinares*
AB282835	*Zalieutes elater*
AB282826	*Lophiodes caulinaris*
AP005978	*Malthopsis jordani*
AB282833	*Chaunax pictus*
AB282838	*Melanocetus johnsonii*
AB282834	*Coelophrys brevicaudata*
AB282845	*Chaenophryne melanorhabdus*
AB282843	*Oneirodes thompsoni*
AB282846	*Bertella idiomorpha*
AB282844	*Puck pinnata*
AB282851	*Ceratias uranoscopus*
AB282850	*Cryptopsaras couesii*
NC_004383	*Caulophryne jordani*
MF994812	*Lophius piscatorius*
KJ020931	*Lophius litulon*

Sources.

1. Stamatakis A. 2014 RAxML version 8: a tool for phylogenetic analysis and post-analysis of large phylogenies. Bioinformatics 30, 1312–1313. (doi:10.1093/bioinformatics/btu033)

2. Martin M. 2011 Cutadapt removes adapter sequences from high-throughput sequencing reads. EMBnet.journal 17, 10-12. (doi:10.14806/ej.17.1.200)

3. Magoc T, Salzberg SL. 2011 FLASH: fast length adjustment of short reads to improve genome assemblies. Bioinformatics 27, 2957–2963. (doi:10.1093/bioinformatics/btr507)

4. Bankevich A et al. 2012 SPAdes: A New Genome Assembly Algorithm and Its Applications to Single-Cell Sequencing. J. Comput. Biol. 19, 455–477. (doi:10.1089/cmb.2012.0021)

5. Gurevich A, Saveliev V, Vyahhi N, Tesler G. 2013 QUAST: quality assessment tool for genome assemblies. Bioinformatics 29, 1072–1075. (doi:10.1093/bioinformatics/btt086)

6. Waterhouse RM, Seppey M, Simão FA, Manni M, Ioannidis P, Klioutchnikov G, Kriventseva EV, Zdobnov EM. 2018 BUSCO Applications from Quality Assessments to Gene Prediction and Phylogenomics. Mol. Biol. Evol. 35, 543–548. (doi:10.1093/molbev/msx319)

7. Marçais G, Kingsford C. 2011 A fast, lock-free approach for efficient parallel counting of occurrences of k-mers. Bioinformatics 27, 764–770. (doi:10.1093/bioinformatics/btr011)

8. Malmstrøm M et al. 2016 Evolution of the immune system influences speciation rates in teleost fishes. Nat. Genet. 48, 1204–1210. (doi:10.1038/ng.3645)

9. Notredame C, Higgins DG, Heringa J. 2000 T-coffee: a novel method for fast and accurate multiple sequence alignment 1 1Edited by J. Thornton. J. Mol. Biol. 302, 205–217. (doi:10.1006/jmbi.2000.4042)

10. Burge C, Karlin S. 1997 Prediction of complete gene structures in human genomic DNA. J. Mol. Biol. 268, 78–94. (doi:10.1006/jmbi.1997.0951)
